# Teen clinics: missing the mark? Comparing pregnancy and sexually transmitted infections rates among enrolled and non-enrolled adolescents

**DOI:** 10.1186/s12939-016-0386-9

**Published:** 2016-06-21

**Authors:** Souradet Y. Shaw, Colleen Metge, Carole Taylor, Mariette Chartier, Catherine Charette, Lisa Lix, Rob Santos, Joykrishna Sarkar, Nathan C. Nickel, Elaine Burland, Dan Chateau, Alan Katz, Marni Brownell, Patricia J. Martens, the PATHS Equity Team, James Bolton, Marni Brownell, Charles Burchill, Elaine Burland, Mariette Chartier, Dan Chateau, Malcolm Doupe, Greg Finlayson, Randall Fransoo, Chun Yan Goh, Milton Hu, Doug Jutte, Alan Katz, Laurence Katz, Lisa Lix, Patricia J. Martens, Colleen Metge, Nathan C Nickel, Colette Raymond, Les Roos, Noralou Roos, Rob Santos, Joykrishna Sarkar, Mark Smith, Carole Taylor, Randy Walld

**Affiliations:** Centre for Global Public Health, University of Manitoba, Winnipeg, Canada; Department of Community Health Sciences, University of Manitoba, Winnipeg, Canada; Centre for Healthcare Innovation, University of Manitoba, Winnipeg, Canada; Manitoba Centre for Health Policy, University of Manitoba, Winnipeg, Canada; Healthy Child Manitoba, Winnipeg, Canada

**Keywords:** School-based clinics, Teen pregnancies, STIs, Out of school youth

## Abstract

**Background:**

In Manitoba, Canada, school-based clinics providing sexual and reproductive health services for adolescents have been implemented to address high rates of sexually transmitted infections (STIs) and pregnancies.

**Methods:**

The objectives of this population-based study were to compare pregnancy and STI rates between adolescents enrolled in schools with school-based clinics, those in schools without clinics, and those not enrolled in school. Data were from the PATHS Data Resource held in the Population Health Research Data Repository housed at the Manitoba Centre for Health Policy. Adolescents aged 14 to 19 between 2003 and 2009 were included in the study. Annualized rates of pregnancies and positive STI tests were estimated and Poisson regression models were used to test for differences in rates amongst the three groups.

**Results:**

As a proportion, pregnancies among non-enrolled female adolescents accounted for 55 % of all pregnancies in this age group during the study period. Pregnancy rates were 2–3 times as high among non-enrolled female adolescents. Compared to adolescents enrolled in schools without school-based clinics, age-adjusted STI rates were 3.5 times (*p* < .001) higher in non-enrolled males and 2.3 times (*p* < .001) higher in non-enrolled females.

**Conclusions:**

The highest rates for pregnancies and STIs were observed among non-enrolled adolescents. Although provision of reproductive and health services to in-school adolescents should remain a priority, program planning and design should consider optimal strategies to engage out of school youth.

**Electronic supplementary material:**

The online version of this article (doi:10.1186/s12939-016-0386-9) contains supplementary material, which is available to authorized users.

## Implications and contribution

Results demonstrate that the highest rates for pregnancies and STIs were observed among non-enrolled adolescents. Parallel strategies to engage out of school youth may potentially impact population-level rates of pregnancies and STIs.

## Background

In North America, the majority of adolescents have experienced sexual intercourse by the time they have reached adulthood [1, 2]. Fostering positive views on sexuality is an important component of development for adolescents as they transition into adulthood; high rates of sexually transmitted infections (STIs) and unwanted pregnancies among adolescents highlight the need for preventive education and health services targeting sexually active adolescents [3–5].

For many adolescents, the school is one of the main sources of information regarding reproductive and sexual health. Consequently, in North America, school-based reproductive and sexual health clinics (“school-based clinics”) have been promoted as a means to deliver health services to adolescents in an accessible manner [6, 7]. However, the literature on the real-world effectiveness of school-based clinics has been mixed, with improvements demonstrated in self-efficacy and knowledge, but not necessarily in actual behaviours [8, 9]. Identification of barriers to and facilitators of access to school-based clinics by adolescents has provided fertile ground for research, with socio-economic status, perceived need, comfort level with staff, physical location, and confidentiality cited as factors influencing the choice to access care [10, 11].

With the increasing focus on school-based clinics, however, there has been less of an emphasis on out-of-school adolescents. Adolescents not engaged in traditional school settings, such as “street-involved youth” (a broad term used to describe youth living or working on the streets) often have disproportionately high rates of STIs [12, 13]. One national study of Canadian street-involved youth found that the relative prevalence of both chlamydia and gonorrhea were 10 and over 20 times higher, respectively, compared with non-street-involved youth [13]. Similarly, engagement with school has been shown to be an important factor in postponing pregnancy [14].

Although these studies suggest that adolescents not enrolled in school may be at higher risk for STIs and early pregnancy, much of the literature on adolescent reproductive health is limited to individuals enrolled in school. Indeed, much of the seminal work in adolescent health uses data where the primary point of contact was in the school. This is because adolescents not enrolled in school are hard to reach and are often missed in adolescent health studies. Limited research exists that directly compares the reproductive health outcomes between in-school adolescents and those not enrolled in school.

In Manitoba, school-based clinics providing adolescent health services are located in community health centres, schools and one hospital. At the time of this study, there were 7 school-based clinics in Winnipeg (the province’s capital city, constituting over half of the province’s total population), and 8 school-based clinics in the province’s rural and northern areas. As part of the criteria for the receipt of funding, schools desiring clinics were asked to justify the need for the clinic, with funding allocated to those schools most “at need”. It is of note that the school-based clinics vary in terms of program characteristics. For example, while all clinics provide free birth control pills, only some provide free contraceptive shots or patches. Some school-based clinics serve high school students while some also middle school students. Also, hours of operation vary widely. At the time of this study, there were 20 clinics located in the community that served both enrolled and non-enrolled adolescents (15 in Winnipeg; 5 in rural and northern areas).

Given the unique opportunity in Manitoba to capture health information on adolescents not enrolled in school, our research objectives were to compare pregnancy and positive STI rates between three groups: adolescents enrolled in schools with school-based clinics, adolescents enrolled in schools without school-based clinics and adolescents who were not enrolled in school. We hypothesized that (1) adolescents not enrolled in school will have higher STI and pregnancy rates than in-school-adolescents, as pregnancy has been shown to be associated with school drop out; and (2) that STI and pregnancy rates in schools with clinics will be higher (lower) than schools without clinics, as clinics were likely located in schools in “higher needs” areas. This study was conducted as part of the PATHS Equity Program of Research, a research program aimed at understanding mechanisms to reduce child health inequity [15].

## Methods

### Data sources

The data for this study are from the PATHS Data Resource held in the Population Health Research Data Repository (the Repository) housed at the Manitoba Centre for Health Policy (MCHP) at the University of Manitoba. The PATHS Resource comprises approximately 99 % of all individuals living in Manitoba, born 1984 to 2012. The Resource includes individual-level health, education and social services administrative data that were originally collected to manage and monitor services. These data contain almost all contacts Manitoba residents have with provincial services throughout childhood, from the prenatal period through to adulthood. The PATHS Resource does not hold personal identifying information, such as names and addresses, but rather an anonymized, scrambled numeric identifier can be used to link individual-level data across files and over time. Thus, researchers are able to construct holistic child health and development trajectories for nearly all children residing in Manitoba. Numerous studies have validated the data within the Resource for research purposes [16–21] and other studies have been published which specifically used the PATHS Resource, to study child health equity [22–24].

The specific data files used in the analyses were:

1. Manitoba Health Insurance Registry, which captures all Manitobans eligible to receive health services and includes demographic information and 6-digit residential postal code for geocoding. Universal health care coverage is offered in Manitoba from a single insurer;
2. Hospital Abstracts, which contain information on all hospitalizations (including birth) in Manitoba and which include up to 16 ICD-9-CM diagnostic codes for discharges before April 1, 2004 and up to 25 ICD10-CA diagnostic codes for discharges on or after April 1, 2004;
3. Medical Services, which contain information on ambulatory physician visits in Manitoba and include a single ICD-9 diagnostic code associated with each visit, coded to the third digit.
4. Cadham Provincial Laboratory, which provides a range of services, including public health laboratory services and reference services for identification and typing of microorganisms (microbiology, serology and parasitology, and virology); requisition/result level data are available at the individual patient level and include clinical information (travel/treatment history, signs and symptoms, specimen information, and reason for test);
5. Statistics Canada Census information, which is used to determine area-level income, with the Manitoba population divided into income quintiles according to average area-level household income, comprising 5 income groupings;
6. Social Assistance and Management Information Network, which includes information on all individuals and families receiving provincial Employment and Income Assistance;
7. Child and Family Services Information system, which include information on all Manitoba children and their families receiving child welfare services, including in-home services and out-of-home placements;
8. Education data, which include Enrollment, Marks and Assessment data for all high school students in Manitoba schools including information on special education needs and funding.

### Study population

The study population consisted of all adolescents (male and female) aged 14 to 19 years of age, who were either enrolled in grades 9 to 12 (or identified as special needs students at a high school level) or not, with continuous health coverage between fiscal years (April 1 to March 31) 2003 to 2010 (*N* = 181,444). Our study population was divided into three groups: (1) adolescents categorized as not enrolled in school (“non-enrolled”, *N* = 32,067), (2) adolescents enrolled in schools that contained a school clinic (SC, *N* = 26,223), and (3) adolescents enrolled in schools without a school clinic (NSC, *N* = 123,154). Adolescents enrolled in school were identified by the enrolment dataset. A list of schools with clinics was provided in consultation with the Healthy Child Manitoba Office. Adolescents enrolled in schools on this list were classified as attending a school with a SC. Teenagers classified as not enrolled in school were those with no enrolment record in a given year, excluding students graduating in the year of interest, or in the years prior to the year of interest. Students in schools that did not have Grade 12 were also excluded, as their inclusion was thought to potentially bias comparisons, given that sexual activity is known to increase as adolescents age, with those in Grade 12 being the most likely to engage in sexual activity [25]. Furthermore, as the published evidence of older adolescents partnering with younger adolescents is strong [26, 27], it was thought that the network dynamics of schools without Grade 12 students could potentially differ greatly from those with Grade 12 students, as they exclude the group most likely to be engaged in sexual activity. Finally, students who transferred schools mid-year were excluded because allocation to one school was not possible; less than 2 % of students transferred mid-year, so the potential impact on results was thought to be minimal.

### Outcome measures & rate calculations

#### Teenage pregnancy & positive STI tests

Pregnancies were defined with a previously published administrative case definition using hospital abstracts [28, 29]. Additional file 1: Table S1 contains the ICD codes (including diagnoses and procedure codes) used to define pregnancies. As all STI tests are performed at the Cadham Provincial Laboratory we were able to define positive STI cases as positive laboratory tests for chlamydia, gonorrhea or syphilis.

### Statistical analyses

#### Rates

Each outcome of interest was used as the numerator for rate calculations. Denominators were the midpoint population of each corresponding one-year age band for the year in question; for pregnancy, only females were included, while for STI tests, both males and females were included. Rates were stratified by group (i.e., SC, NSC and non-enrolled), and crude pregnancy and STI rates were generated, along with their 95 % confidence intervals (95 % CIs). Rates were also age-adjusted using a generalized linear modelling approach with a Poisson distribution selected, with age (and its quadratic term) and enrolment status entered as covariates, with the entire cohort population used as the standard [30]. Except where indicated, age-adjusted rates are reported. Rates were age-adjusted due to the differences in age structure between the three groups of interest. Rates were not adjusted for income quintile (i.e., an indicator of socio-economic status) as in this instance, socio-economic status likely acts as an effect modifier, rather than a confounder. To address this, the association between enrolment status and the outcome variables were stratified by income quintile.

The groups were compared on a number of socio-demographic and school-related variables. For the purposes of this comparison, information from adolescents in the 2008/09 academic year is presented. The following variables were used in the descriptive analysis: age (in years), sex, current grade, Grade 9 Performance Index, region, income quintile, receipt of income assistance, currently receiving child welfare services, and history of receiving child welfare services. The Grade 9 Performance Index is a standardized, scaled logit measure developed by MCHP researchers that measures the academic performance of students in grade 9, relative to their peers [31]. The Performance Index is calculated using all possible average marks in all classes and the number of credits earned during the grade 9 school year; higher scores on the Index translate to better performance in grade 9. The Index ranged from −2.5 to 2.3 in our cohort. Region was classified into the five Manitoba Regional Health Authorities: Interlake-Eastern, Northern, Eastern, Prairie Mountain and Winnipeg. Similar to previous research, Winnipeg was further divided into three regions by aggregate health status: most, least and average health status [32]. Health status was determined by premature mortality at the neighbourhood cluster (an administrative unit used by the Winnipeg Regional Health Authority) level. Income quintile, an area-level measure of household income based on Statistics Canada dissemination areas, was derived by dividing the population of Manitoba into 5 income groups, so that 20 % of the population is in each group [33]. Receipt of income assistance measures whether or not the individual, or the individual’s family (if under the age of 18) was currently receiving income assistance. Finally, current and historic involvement with child welfare services measures whether or not the adolescent is in or has been in out-of-home care or their family is currently or has historically received protection or support services from the child welfare system in Manitoba [34].

Rates were estimated for groups and income quintiles within groups using a generalized linear model with a Poisson distribution. Relative risks (RR) and 95 % CIs are reported. Model fit was assessed using the ratio of the deviance to the model degrees of freedom; a value close to one indicates a well-fitting model. All analyses were performed using SAS® version 9.3. As this was a study based on de-identified administrative data, informed consent was not obtained. This study was approved by the Health Research Ethics Board at the University of Manitoba and the Health Information Privacy Committee of Manitoba.

## Results

### Socio-demographic characteristics in 2008

The socio-demographic and school related characteristics for adolescents for the three groups in academic year 2008/09 are displayed in Table 1. There were substantial differences in age structure between the three groups; over 50 % of the non-enrolled group was composed of 18 and 19 year olds, compared to approximately 10 % of both the SC and NSC groups. The non-enrolled group also scored lower on the Grade 9 Performance Index, relative to the SC and NSC groups, and were the most likely to reside (40 %) in an area in the lowest income quintile, currently receiving child welfare services (4.5 %), and have a history of involvement with child welfare services (41 %).

**Table 1:** Select socio-demographic and school-related characteristics, youth and adolescents from schools with and without school clinic access and non-enrolled status, 2008/09 academic year (*N* = 66,539)

		Enrolled				Non-Enrolled	
		School clinics (*N* = 9,291)		No school clinics (*N* = 44,924)		(*N* = 12,324)	
		N	%	N	%	N	%
Age (years)	14	1180	12.7	8081	18.0	1282	10.4
	15	2382	25.6	10787	24.0	1223	9.9
	16	2373	25.5	10868	24.2	1414	11.5
	17	2386	25.7	10874	24.2	1921	15.6
	18	721	7.8	3015	6.7	2948	23.9
	19	249	2.7	1299	2.9	3536	28.7
Gender	Male	4739	51.0	22957	51.1	6796	55.1
	Female	4552	49.0	21967	48.9	5528	44.9
Current Grade of Student	9	1655	17.8	10018	22.3		
	10	2665	28.7	11513	25.6		
	11	2395	25.8	10875	24.2		
	12	2428	26.1	12171	27.1		
	Special Needs	148	1.6	347	0.8		
Completion in a Timely Manner	Yes	5604	76.5	30509	81.1	1393	38.3
	No	1720	23.5	7115	18.9	2248	61.7
Grade 9 Performance Index	N [Mean (SD^a^)]	7410	[−0.22 (1.0)]	37808	[0.06 (0.0)]	3889	[−1.13 (1.0)]
Regions	Interlake-Eastern	1197	12.9	3818	8.5	1663	13.5
	Northern	1714	18.5	2209	4.9	2661	21.6
	Southern	964	10.4	8350	18.6	2303	18.7
	Prairie Mountain	61	0.7	4347	9.7	1383	11.2
	Winnipeg Average Healthy	317	3.4	6424	14.3	773	6.3
	Winnipeg Least Healthy	2879	31.0	5569	12.4	2118	17.2
	Winnipeg Most Healthy	2159	23.2	14207	31.6	1423	11.6
Income Quintile (IQ)	NF^b^	184	2.0	534	1.2	258	2.1
	Q1^b^	2033	21.9	6910	15.4	4947	40.1
	Q2^b^	1756	18.9	8395	18.7	2493	20.2
	Q3^b^	1173	12.6	9420	21.0	2102	17.1
	Q4^b^	1877	20.2	9521	21.2	1429	11.6
	Q5^b^	2268	24.4	10144	22.6	1095	8.9
Income Assistance	Yes	1250	13.5	2793	6.2	1505	12.2
	No	8041	86.6	42131	93.8	10819	87.8
Current in Protective Services	Yes	307	3.3	781	1.7	556	4.5
	No	8984	96.7	44143	98.3	11768	95.5
History of Protective Services	Yes	3150	33.9	9921	22.1	5093	41.3
	No	6141	66.1	35003	77.9	7231	58.7

^a^ *SD* standard deviation, ^b^ *NF* not found, *Q1* quintile 1 (lowest income quintile), *Q2* quintile 2, *Q3* quintile 3, *Q4* quintile 5 (highest income quintile)

The remaining results section focuses on the 181,444 adolescents included in the multiple years available for this study, of which 14 % (26,223/181,444) were SC, 68 % (123,154/181,444) were NSC and 18 % (32,067/181,444) were non-enrolled youth.

### Pregnancy

From 2003 to 2009 a total of 9,292 pregnancies were recorded in the cohort of adolescent females, with over 55 % (5,140/9,292) occurring among those in the non-enrolled group (Table 2). Pregnancies in SC females aged 14 to 19 accounted for approximately 10 % of all pregnancies in the sample during this time period, for an age-adjusted pregnancy rate for SC females aged 14 to 19 of 42.8 per 1000. The pregnancy rate for NSC females was 31.8 per 1,000 and 87.9 per 1,000 for those females not enrolled. The rate for non-enrolled females was 2.1 times (*p* < .0001) higher than SC females and 2.8 times (*p* < .0001) higher than NSC females (Table 3). Crude rates and relative rates by income quintile are available in Additional file 1: Tables S2 and S3.

**Table 2:** Crude and age-adjusted pregnancy and sexually transmitted infections rates, by enrolled/non-enrolled group, 2000–2009

	School clinic (SC)	No school clinic (NSC)	Non-Enrolled	Total
Pregnancy				
No.	939	3213	5140	9292
Crude rate (per 1,000)	35.8 (33.5–38.1)	26.1 (25.2–27.0)	160.3 (155.9–164.7)	51.2 (50.2–52.3)
Age-adjusted	42.8 (40.0–45.8)	31.8 (30.5–33.1)	87.9 (84.8–91.1)	53.3 (52.2–54.5)
STIs				
Female				
No.	467	1134	1112	2713
Crude rate (per 1,000)	17.8 (16.2–19.4)	9.2 (8.7–9.7)	34.7 (32.6–36.7)	15.0 (14.4–15.5)
Age-adjusted	19.5 (17.7–21.5)	10.2 (9.5–11.0)	23.9 (22.2–25.7)	16.5 (15.9–17.1)
Male				
No.	200	449	935	1584
Crude rate (per 1,000)	7.1 (6.1–8.1)	3.5 (3.1–3.8)	23.3 (21.8–24.7)	8.0 (7.6–8.4)
Age-adjusted	8.3 (7.1–9.6)	4.1 (3.7–4.6)	14.3 (13.1–15.6)	7.8 (7.4–8.3)
Total				
No.	667	1583	2047	4297
Crude rate (per 1,000)	12.3 (11.3–13.2)	6.3 (5.9–6.6)	28.3 (27.1–29.6)	11.3 (11.0–11.7)
Age-adjusted	13.7 (12.6–14.9)	7.1 (6.7–7.5)	18.9 (17.9–20.0)	12.1 (11.7–12.5)

**Table 3:** Relative Rates (RR) and 95 % confidence intervals (95 % CI) of Crude and Age-adjusted Rates, Non-enrolled group

	RR (95 % CI)		RR (95 % CI)		RR (95 % CI)
Pregnancy					
Non-enrolled vs. SC^a^ (crude)	4.5 (4.2–4.8)	Non-enrolled vs. NSC^a^ (crude)	6.1 (5.9–6.4)	SC vs. NSC^a^ (crude)	1.4 (1.3–1.5)
Non-enrolled vs. SC^a^ (adjusted)	2.1 (1.9–2.2)	Non-enrolled vs. NSC^a^ (adjusted)	2.8 (2.6–2.9)	SC vs. NSC^a^ (adjusted)	1.3 (1.3–1.4)
STIs					
Female					
Non-enrolled vs. SC^a^ (crude)	1.9 (1.7–2.2)	Non-enrolled vs. NSC^a^ (crude)	3.8 (3.5–4.1)	SC vs. NSC^a^ (crude)	1.9 (1.7–2.2)
Non-enrolled vs. SC^a^ (adjusted)	1.2 (1.1–1.4)	Non-enrolled vs. NSC^a^ (adjusted)	2.3 (2.1–2.6)	SC vs. NSC^a^ (adjusted)	1.9 (1.7–2.1)
Male					
Non-enrolled vs. SC^a^ (crude)	3.3 (2.8–3.8)	Non-enrolled vs. NSC^a^ (crude)	6.7 (6.0–7.5)	SC vs. NSC^a^ (crude)	2.1 (1.7–2.4)
Non-enrolled vs. SC^a^ (adjusted)	1.7 (1.5–2.0)	Non-enrolled vs. NSC^a^ (adjusted)	3.5 (3.1–3.9)	SC vs. NSC^a^ (adjusted)	2.0 (1.7–2.4)
Total					
Non-enrolled vs. SC^a^ (crude)	2.3 (2.1–2.5)	Non-enrolled vs. NSC^a^ (crude)	4.5 (4.2–4.8)	SC vs. NSC^a^ (crude)	2.0 (1.8–2.2)
Non-enrolled vs. SC^a^ (adjusted)	1.4 (1.3–1.5)	Non-enrolled vs. NSC^a^ (adjusted)	2.7 (2.5–2.9)	SC vs. NSC^a^ (adjusted)	1.9 (1.7–2.2)

^a^ *SC* schools with clinics, *NSC* schools without clinics

#### Income quintile

Regardless of school clinic or enrollment status, a steep gradient, by income quintile, was observed in pregnancy rates (Table 4). Generally speaking, low-income areas had the highest pregnancy rates, while the lowest pregnancy rates were observed in high-income areas. At 134.4 per 1,000, the highest pregnancy rate was observed among non-enrolled females from the lowest income quintile areas (i.e., Q1 residents). Among Q1 residents, the pregnancy rate for non-enrolled females was 1.7 times (*p* < .0001) higher than SC females and 2.0 times (*p* < .0001) higher than NSC females (Table 5).

**Table 4:** Age-adjusted pregnancy and sexually transmitted infections rates per 1000 teens ages 15–19, by enrolled/non-enrolled group and income quintile, 2000–2009

Neighbourhood income quintile	Age-adjusted rate per 1000 School Clinic (SC)	Age adjusted rate per 1000 No School Clinic (NSC)	Age-adjusted rate per 1000 Non-Enrolled	Overall age-adjusted rate per 1000 Total
Pregnancy^a^				
Q1 (lowest)	76.8 (69.7–84.6)	66.9 (63.3–70.8)	134.4 (129.2–139.9)	99.39 (96.37–102.51)
Q2	44.4 (38.2–51.6)	38.8 (36.1–41.7)	96.5 (90.8–102.5)	58.60 (56.07–61.23)
Q3	34.4 (28.1–42.2)	28.1 (25.8–30.5)	78.5 (72.5–85.0)	41.70 (39.47–44.04)
Q4	32.8 (27.7–38.8)	20.3 (18.4–22.3)	57.5 (51.8–63.8)	29.67 (27.79–31.67)
Q5 (highest)	24.3 (20.5–28.9)	11.7 (10.3–13.3)	51.1 (45.1–57.9)	20.18 (18.66–21.84)
STIs^a^				
Female				
Q1 (lowest)	43.4 (38.2–49.4)	25.2 (23.0–27.7)	42.2 (39.0–45.6)	34.5 (32.7–36.4)
Q2	20.2 (16.2–25.1)	14.3 (12.7–16.0)	27.5 (24.2–31.2)	18.5 (17.1–20.0)
Q3	12.5 (9.0–17.4)	7.9 (6.8–9.2)	16.8 (13.8–20.4)	10.1 (9.0–11.3)
Q4	11.7 (8.9–15.4)	6.0 (5.0–7.1)	13.0 (10.1–16.7)	7.9 (6.9–8.9)
Q5 (highest)	11.1 (8.7–14.1)	3.6 (2.9–4.5)	10.9 (8.0–14.8)	5.8 (5.0–6.7)
Male				
Q1 (lowest)	18.0 (14.8–21.8)	11.6 (10.2–13.3)	24.0 (22.0–26.3)	18.3 (17.1–19.7)
Q2	8.7 (6.4–11.8)	5.2 (4.3–6.2)	13.4 (11.5–15.5)	8.5 (7.6–9.4)
Q3	5.8 (3.7–9.0)	2.2 (1.7–2.9)	9.4 (7.7–11.6)	4.5 (3.8–5.2)
Q4	3.6 (2.3–5.8)	1.4 (1.0–1.9)	7.3 (5.7–9.4)	3.0 (2.5–3.6)
Q5 (highest)	2.9 (1.8–4.6)	1.2 (0.9–1.8)	6.9 (5.1–9.2)	2.5 (2.1–3.1)

^a^ *Q1* quintile 1 (lowest income quintile), *Q2* quintile 2, *Q3* quintile 3, *Q4* quintile 5 (highest income quintile)

**Table 5:** Relative Rates (RR) and 95 % confidence intervals (95 % CI), non-enrolled youth, vs. SC and NSC youth, by income quintile (age-adjusted)

	RR of non-enrolled vs. School Clinic (SC) as reference	RR of non-enrolled vs. No School Clinic (NSC) as reference
Pregnancy^a^		
RR (95 % CI): Q1	1.7 (1.6–1.9)	2.0 (1.9–2.1)
RR (95 % CI): Q2	2.2 (1.8–2.5)	2.5 (2.3–2.7)
RR (95 % CI): Q3	2.3 (1.8–2.8)	2.8 (2.5–3.1)
RR (95 % CI): Q4	1.8 (1.4–2.2)	2.9 (2.5–3.3)
RR (95 % CI): Q5	2.1 (1.7–2.6)	4.4 (3.7–5.3)
STIs^a^		
Female		
RR (95 % CI): Q1	1.0 (0.8–1.1)	1.7 (1.5–1.9)
RR (95 % CI): Q2	1.4 (1.1–1.8)	1.9 (1.6–2.3)
RR (95 % CI): Q3	1.3 (0.9–2.0)	2.1 (1.7–2.7)
RR (95 % CI): Q4	1.1 (0.8–1.6)	2.2 (1.6–3.0)
RR (95 % CI): Q5	1.0 (0.7–1.5)	3.0 (2.1–4.4)
Male		
RR (95 % CI): Q1	1.3 (1.1–1.7)	2.1 (1.8–2.4)
RR (95 % CI): Q2	1.5 (1.1–2.2)	2.6 (2.0–3.3)
RR (95 % CI): Q3	1.6 (1.0–2.7)	4.3 (3.0–6.0)
RR (95 % CI): Q4	2.0 (1.2–3.4)	5.3 (3.5–8.0)
RR (95 % CI): Q5	2.4 (1.4–4.1)	5.5 (3.5–8.8)
Total		
RR (95 % CI): Q1	1.1 (1.0–1.2)	1.8 (1.6–2.0)
RR (95 % CI): Q2	1.4 (1.2–1.7)	2.1 (1.8–2.4)
RR (95 % CI): Q3	1.5 (1.1–2.0)	2.6 (2.2–3.2)
RR (95 % CI): Q4	1.3 (1.0–1.8)	2.8 (2.2–3.6)
RR (95 % CI): Q5	1.3 (1.0–1.7)	3.7 (2.8–5.0)

^a^ *Q1* quintile 1 (lowest income quintile), *Q2* quintile 2, *Q3* quintile 3, *Q4* quintile 5 (highest income quintile)

### STIs

From 2003 to 2009, a total of 4,297 positive STI tests were reported for the cohort of adolescents for an overall rate of 12.1 per 1,000 (Table 2). At 16.5 per 1,000, female rates were over twice as high as male rates (7.8 per 1,000). Approximately 48 % (2,047/4,297) of all STIs were reported from members of the non-enrolled group. STI rates were highest in the non-enrolled group for both males (14.3 per 1,000) and females (23.9 per 1,000), and lowest in the NSC group. Compared to the NSC group, the adjusted rate in the non-enrolled group was 3.5 times (*p* < .0001) higher in males, and 2.3 times (*p* < .0001) higher in females (Table 3).

#### Income quintile

Similar to pregnancy rates, a gradient was observed by income quintile, with the highest STI rates in the lowest income quintile areas, and the lowest STI rates in the highest income areas (Table 4). Generally speaking, a statistically significant increase in rates was observed when comparing the non-enrolled group to the SC and NSC groups, even when stratified by income quintile (Table 5), and by sex. For example, among males living in areas with the lowest income (i.e., Q1), positive STI tests were 2.1 times (*p* < .0001) higher in the non-enrolled group, compared to the NSC group. Of some interest, and for both pregnancy and STIs, the discrepancy between non-enrolled youth and either SC or NSC youth increased as a function of income quintile.

## Discussion

Our results demonstrate that the highest rates of pregnancy and STIs were found among youth not enrolled in schools. Compared to youth enrolled in schools without clinics, youth not enrolled in schools had almost three times the rate of pregnancies and STIs. The higher rates observed in non-enrolled youth were observed even after stratifying by area-level wealth, although the association was more pronounced for pregnancy and STIs among males. As part of the criteria for the receipt of funding, schools desiring clinics were asked to justify the need for the clinic, with funding allocated to those schools most “at need”. Thus, high-risk schools were targeted by the school-based clinic program, consistent with recommendations from the literature [35]. To the best of our knowledge, ours is the first population-based study to explicitly compare rates of pregnancy and STIs among youth not enrolled in schools, to those youth enrolled in schools with, and without school-based clinics in Canada. The results from this study suggest program implementers were successful in their targeting efforts, as among those enrolled in schools, youth from schools with clinics were at highest need, irrespective of indicator examined. At the same time, our results indicate that out-of-school youth accounted for the majority of teen pregnancies and STIs. Thus, taken together, these results stress the urgent need for prevention and intervention services aimed at out-of-school youth, alongside strategies that provide accessible care to youth attending schools.

Generally speaking, the finding that youth who were not enrolled in school had the poorest outcomes in our study is consistent with the literature [14, 36]. Among a sample of high-risk African American girls, Crosby et al. demonstrated that girls who dropped out of school were two times as likely to test positive for STIs (specifically, *Trichomonas vaginalis* and/or *Chlamydia trachomatis*)*,* compared to those who remained enrolled in school [36]. It should be noted that some studies have demonstrated evidence of school-based clinics being effective in producing positive academic outcomes [37], including improving attendance, and modest reductions in school dropout rates among moderate users of school clinics [37–39]. Moreover, research has shown that even among those already pregnant, the provision of prenatal care reduced school absenteeism and dropout rates [40]. Given evidence suggesting consistent school attendance as a protective factor in reducing adolescent pregnancies [14], the provision of school-based health care can potentially work in a preventative manner to delay or reduce school dropout rates, and can also work in concert with other services that more formally target out-of-school youth, ultimately providing a comprehensive set of services for youth. In terms of policy implications, although the focus of funding in Manitoba was for school-based health clinics, our findings suggest that to see an impact at the population level, programs that engage out-of-school youth through outreach and/or tailored programming need also be considered alongside school-based clinics. Moreover, in addition to medical services, there is also potential for the school-based clinics to either develop, or partner with organizations that offer other services, such as prenatal care.

Our study has a number of strengths. By using population-based data on nearly all adolescents, we were able to include individuals from marginalized populations which are frequently not captured using survey data (15; 17-20); this increases the generalizability of our findings vis-à-vis health equity research. Using administrative data allowed us to both leverage objective measures of the outcomes used in the study and avoid the problems associated with recall bias (18-20). In spite of the several strengths of our study, there are limitations worth noting. Although we were able to adjust for an expansive number of measured confounders, we were unable to account for unmeasured confounding, as well; as such, we cannot draw inferences about the causal impact of school clinics on STI rates from our results. A related limitation was our inability to identify the mechanisms that were driving the statistically significant differences we found, which was beyond the scope of this manuscript. As well, because we do not have site-specific data describing the operation and school environment for each clinic, we were unable to explore how variations in clinic operations may have impacted our results.

## Conclusions

Our results demonstrate that given their high STI and pregnancy rates, out-of-school youth are an important group to target, with respect to the provision of reproductive health services. Because of the differences in outcome measures found between those schools which have school-based clinics and those that do not, and because the school-based clinics vary from school to school (for example, in number of hours of operation) our comparisons cannot determine if the school based clinics have had an impact on the rates of STIs or pregnancies. In addition to the mixed evidence regarding whether or not provision of reproductive health services within schools can have an impact on reproductive health outcomes, further studies are needed on the effectiveness of youth-oriented clinics across a variety of school and community settings and other interventions to engage youth not involved with the school system. As well, research into understanding the trajectory of youth who fall out of the school system is also necessary, thus building better predictive models to inform interventions designed to engage with youth prior to dropping out of the school system. A holistic, comprehensive and systematic approach to prevention and intervention reproductive health services, with linkages among school-based clinics, community based teen clinics, and other outreach services for out of school youth should be emphasised.

## Abbreviations

95 % CI, 95 % confidence intervals; ICD, International Classification of Diseases; MCHP, Manitoba Centre for Health Policy; NSC, no school clinic; SC, school clinic; STI, sexually transmitted infections

## Additional file

Additional file 1:
**Table S1:** ICD-9 and ICD-10 codes used to define pregnancies. **Table S2:** Crude pregnancy and sexually transmitted infections rates, by enrolled/non-enrolled group and income quintile, 2000-2009. **Table S3:** Relative Rates of non-enrolled, by Income Quintile (Crude). (DOCX 18 kb)
