# Supplementary material for: Teen clinics: missing the mark? Comparing pregnancy and sexually transmitted infections rates among enrolled and non-enrolled adolescents
Source: Int J Equity Health. 2016 Jun 21;15:95. doi: 10.1186/s12939-016-0386-9 (PMC4915138; doi:10.1186/s12939-016-0386-9)
Supplement: Additional file 1: — Table S1: ICD-9 and ICD-10 codes used to define pregnancies. Table S2: Crude pregnancy and sexually transmitted infections rates, by enrolled/non-enrolled group and income quintile, 2000-2009. Table S3: Relative Rates of non-enrolled, by Income Quintile (Crude). (DOCX 18 kb) [file 12939_2016_386_MOESM1_ESM.docx]

**Table S1: ICD-9 and ICD-10 codes used to define pregnancies**

|  | Code | Description |
| --- | --- | --- |
| ICD-9-CM (up to 2004) | V27 | Outcome of delivery |
|  | 632 | Missed abortion |
|  | 633 | Ectopic pregnancy |
|  | 634 | Spontaneous abortion |
|  | 635 | Legally induced abortion |
|  | 636 | Illegally induced abortion |
|  | 637 | Unspecified abortion |
|  | 656.4 | Intrauterine death |
| ICD-10-CA (after 2004) | Z37 | Delivery |
|  | O02.1 | Missed abortion |
|  | O03 | Spontaneous abortion |
|  | O04 | Medical abortion |
|  | O05 | Other abortion |
|  | O06 | Failed attempted abortion |
|  | O07 | Complications following abortion and ectopic and molar pregnancy |
|  | O36 | Maternal care for other known or suspected fetal problems |
| Procedure Codes | 66.62 | Salpingectomy with removal of tubal pregnancy |
|  | 69.01 | Dilation and curettage for termination of pregnancy |
|  | 69.51 | Aspiration curettage of uterus for termination of pregnancy |
|  | 74.3 - CCI code 5.CA.93 | Removal of extratubal ectopic pregnancy |
|  | 74.91 - CCI codes 5.CA.89, 5.CA.90 | Hysterotomy to terminate pregnancy |
|  | 75.0 – CCI code 5.CA.88, CCI code 5.MD.5 or 5.MD.60 | Intra-amniotic injection for abortion |

**Table S2: Crude pregnancy and sexually transmitted infections rates, by enrolled/non-enrolled group and income quintile, 2000-2009**

| **Crude rate** | **School Clinic (SC)** | **No School Clinic (NSC)** | **Non-Enrolled** | **Total** |
| --- | --- | --- | --- | --- |
| Pregnancy | | | | |
| Q1 (lowest) | 69.3 (62.6-76.0) | 62.4 (58.9-65.8) | 209.0 (201.2-216.8) | 113.2 (109.9-116.5) |
| Q2 | 36.9 (31.4-42.5) | 32.4 (30.0-34.7) | 156.7 (147.5-165.8) | 58.3 (55.8-60.8) |
| Q3 | 27.8 (22.1-33.4) | 22.2 (20.4-24.0) | 129.5 (119.4-139.5) | 38.2 (36.1-40.3) |
| Q4 | 25.6 (21.3-29.9) | 15.5 (14.0-17.0) | 95.3 (85.5-105.0) | 25.6 (24.0-27.3) |
| Q5 (highest) | 18.5 (15.4-21.7) | 8.6 (7.5-9.7) | 85.3 (74.7-95.8) | 16.4 (15.1-17.7) |
| STIs | | | | |
| Female | | | | |
| Q1 (lowest) | 39.2 (34.2-44.2) | 23.1 (21.0-25.2) | 50.3 (46.5-54.2) | 34.8 (32.9-36.6) |
| Q2 | 17.6 (13.8-21.4) | 12.4 (11.0-13.8) | 33.7 (29.4-38.0) | 17.4 (16.0-18.8) |
| Q3 | 10.8 (7.2-14.3) | 6.7 (5.7-7.7) | 20.8 (16.8-24.9) | 9.1 (8.1-10.1) |
| Q4 | 9.9 (7.2-12.6) | 5.0 (4.1-5.8) | 16.2 (12.2-20.2) | 6.9 (6.1-7.8) |
| Q5 (highest) | 9.3 (7.0-11.5) | 3.0 (2.3-3.6) | 13.6 (9.4-17.8) | 5.0 (4.3-5.7) |
| Male | | | | |
| Q1 (lowest) | 16.9 (13.6-20.1) | 11.1 (9.6-12.6) | 34.9 (32.0-37.8) | 20.9 (19.5-22.3) |
| Q2 | 7.8 (5.4-10.1) | 4.6 (3.7-5.5) | 20.8 (17.8-23.8) | 8.8 (7.9-9.8) |
| Q3 | 5.1 (2.8-7.4) | 1.9 (1.4-2.4) | 15.1 (12.0-18.1) | 4.4 (3.7-5.1) |
| Q4 | 3.1 (1.7-4.6) | 1.2 (0.8-1.6) | 11.7 (8.8-14.7) | 2.9 (2.3-3.4) |
| Q5 (highest) | 2.5 (1.3-3.6) | 1.0 (0.7-1.4) | 11.2 (7.9-14.5) | 2.3 (1.8-2.7) |

**Table S3: Relative Rates of non-enrolled, by Income Quintile (Crude)**

|  | **School Clinic as reference** | **No School Clinic as reference** |
| --- | --- | --- |
| **Pregnancy** |  |  |
| RR (95%CI): Q1 | 3.0 (2.7-3.3) | 3.4 (3.1-3.6) |
| RR (95%CI): Q2 | 4.2 (3.6-5.0) | 4.8 (4.4-5.3) |
| RR (95%CI): Q3 | 4.7 (3.7-5.8) | 5.8 (5.2-6.5) |
| RR (95%CI): Q4 | 3.7 (3.1-4.5) | 6.1 (5.3-7.1) |
| RR (95%CI): Q5 | 4.6 (3.7-5.7) | 9.9 (8.3-11.8) |
| **STIs** |  |  |
| Female |  |  |
| RR (95%CI): Q1 | 1.3 (1.1-1.5) | 2.2 (1.9-2.5) |
| RR (95%CI): Q2 | 1.9 (1.5-2.5) | 2.7 (2.3-3.2) |
| RR (95%CI): Q3 | 1.9 (1.3-2.8) | 3.1 (2.4-4.0) |
| RR (95%CI): Q4 | 1.6 (1.1-2.4) | 3.2 (2.4-4.4) |
| RR (95%CI): Q5 | 1.5 (1.0-2.2) | 4.6 (3.1-6.7) |
| Male |  |  |
| RR (95%CI): Q1 | 2.1 (1.7-2.6) | 3.1 (2.7-3.7) |
| RR (95%CI): Q2 | 2.7 (1.9-3.8) | 4.5 (3.6-5.7) |
| RR (95%CI): Q3 | 2.9 (1.8-4.8) | 7.9 (5.6-11.1) |
| RR (95%CI): Q4 | 3.7 (2.2-6.3) | 10.0 (6.6-15.2) |
| RR (95%CI): Q5 | 4.5 (2.6-7.8) | 11.1 (7.0-17.6) |
| Total |  |  |
| RR (95%CI): Q1 | 1.5 (1.3-1.7) | 2.5 (2.2-2.7) |
| RR (95%CI): Q2 | 2.1 (1.8-2.6) | 3.1 (2.7-3.6) |
| RR (95%CI): Q3 | 2.3 (1.7-3.0) | 4.2 (3.4-5.0) |
| RR (95%CI): Q4 | 2.1 (1.6-2.9) | 4.6 (3.6-5.7) |
| RR (95%CI): Q5 | 2.1 (1.5-2.8) | 6.2 (4.7-8.3) |
